# Modulation of ZnT-1 by Let7a unveils a therapeutic potential in amyotrophic lateral sclerosis

**DOI:** 10.1016/j.neurot.2025.e00571

**Published:** 2025-03-19

**Authors:** Serenella Anzilotti, Cristina Franco, Valeria Valsecchi, Ornella Cuomo, Giovanna Lombardi, Noemi Di Muraglia, Nunzia De Iesu, Giusy Laudati, Lucio Annunziato, Lorella Maria Teresa Canzoniero, Giuseppe Pignataro

**Affiliations:** a Department of Human Sciences and Quality of Life Promotion, San Raffaele University, 00166 Rome, Italy; b Department of Science and Technology, University of Sannio, Benevento, Italy; c Division of Pharmacology, Department of Neuroscience, Reproductive and Dentistry Sciences, School of Medicine, University of Naples Federico II, Naples, Italy; d International School of Advanced Studies, University of Camerino, Camerino, Italy; e IRCCS Synlab SDN S.p.A, Naples, Italy

**Keywords:** ALS, G93A, microRNA, ZnT1

## Abstract

The imbalance in cellular ionic homeostasis represents a hallmark of several neurodegenerative diseases, including Amyotrophic Lateral Sclerosis (ALS). Zinc Transporter 1 (ZnT1), the first described member of the ZnT family, stands out as the sole member of the SLC30 family responsible for exporting cytosolic zinc to the extracellular space. While ZnT1 is expressed across all tissues and cell types studied, it exhibits the highest prominence within the central nervous system. In ALS SOD1^G93A^ mice, a reduction in ZnT1 expression consistent with disease progression has been observed, prompting our investigation into its role in ALS pathophysiology. Remarkably, through the use of a sequence complementary to the microRNA let-7a (anti-Let-7a) able to modulate ZnT1 expression, we demonstrated in ALS mice its capability to: (1) prevent the reduction in ZnT1 levels in the spinal cord; (2) preserve motor neuron survival in the ventral spinal horn; (3) decrease astroglial and microglial activation while sparing resident microglial cells in the spinal cord; and (4) improve the lifespan and alleviate motor symptoms.

## Introduction

Motor neuron (MN) diseases represent a pharmacological medical need, since no effective treatments are yet available. Among this group of disorders, Amyotrophic Lateral Sclerosis represents one of the most devasting disease. In fact, people affected by ALS die within 30 months of symptoms onset [1] and show an increase in the average survival rate of 10–16 weeks after the use of the pharmacological treatment now available. The death usually is due to MN loss in the spinal cord and brain, followed by muscle paralysis [2]. More than 90 ​% of ALS cases are sporadic, however, several genes have been associated with the familiar forms of ALS. Among them, the gene encoding for the copper-zinc superoxide dismutase, SOD1, is the best characterized [3]. In the neurodegenerative mechanisms associated with the SOD1 mutation, a pivotal role seems to be played by the disrupted ionic homeostasis [4,5]. Notably, since 3−10 ​% of human genes encode Zn-containing proteins, this metal seems to play an important role in the correct functioning of numerous proteins [6,7]. In fact, the disruption of zinc homeostasis has been associated to numerous human disorders, including growth retardation, dermatological injuries, and neurological disorders [[8], [9], [10]]. Intracellular and extracellular zinc homeostasis is regulated by Zn transporters and metallothioneins (MTs). In particular, the ZnT/SLC30 transporter family consists of 10 members, all known to exhibit six transmembrane domains, a conserved domain between transmembrane tracts 4 and 5 rich in histidine/serine and the amino- and carboxy-terminal ends located intracellularly [11]. ZnT1, the first member of the ZnT family to be identified, is ubiquitously expressed in all tissues and cell types and represents a primary route for Zn^2+^ efflux in neurons [[12], [13], [14], [15], [16]]. It has been demonstrated that Zinc transporters ZnT3 and ZnT6 are downregulated in the spinal cord of patients with sporadic amyotrophic lateral sclerosis [17]. In ALS mice, a reduction in ZnT1 expression consistent with disease progression has been observed, prompting our investigation into its role in ALS pathophysiology. To this aim, we have firstly identified, through in silico analysis, a miRNA, Let-7a, capable of determining a downregulation of ZnT1 expression. This microRNA is implicated in numerous neurodegenerative diseases such as Alzheimer's [18], Parkinson's [19], and cerebral ischemia [20], and finally, it is also a therapeutic target in nerve regeneration [21].

For this reason, to investigate the role of ZnT1 in ALS, we used SOD1^G93A^ mice, a familial model of ALS, to determine: (i) mRNA and protein expression of ZnT1 in the spinal cord of mice at different disease stages, (ii) microRNAs capable of regulating ZnT1 expression, (iii) whether ZnT1 modulation through microRNAs can improve motor neuron survival, reduce neuroinflammation, and ameliorate motor performance.

## Materials and methods

### Reagents

All culture materials were purchased from Gibco® (Life Technologies, Italy) unless otherwise indicated. The Retinoic acid (RA), L-BMAA(S(+)-2-Amino-3-(methylamino)propionic acid hydrochloride), 3-[4,5-dimethylthiazol-2-yl]-2,5-diphenyltetrazolium bromide (MTT), Protease Inhibitor Cocktail were from Merck Life Science (Milan, Italy). RA was dissolved in DMSO and stock solutions were prepared to reach a final concentration of 0.1 ​% of the above solvent. ZnT1 and Negative control siRNAs, Let-7a Mimic, anti-Let-7a-5p miRNA inhibitor, and the corresponding negative control (NT-miR), and HiPerFect Transfection Reagent were purchased from©Qiagen (Milan, Italy)

### Cell cultures and treatment

The rat pheochromocytoma cells line PC12 was cultured in RPMI-1640 medium containing 10 ​% heat-inactivated Horse Serum (HS), 5 ​% Fetal Bovine Serum (FBS), 2 ​mM l-glutamine, 50 IU/ml penicillin and 50 ​μg/ml streptomycin in humidified air with 5 ​% CO_2_. Cells were plated in 60 ​mm Petri dishes 24 ​h before transfection. The motor neuron-like hybrid cell line NSC-34 was cultured in Dulbecco's Modified Eagle's Medium (DMEM), 10 ​% heat-inactivated FBS, 2 ​mM l-glutamine and 50 IU/ml penicillin and 50 ​μg/ml streptomycin at 37 ​°C in humidified air with 5 ​% CO_2_. The maximum number of passages was 34. For differentiation, cells were seeded for 48 ​h in 12-well plates and then switched to DMEM/Ham's F12 Eagle's medium (1:1) supplemented with 1 ​% non-essential amino acid solution (NEAA), 1 ​% FBS, 50 IU/ml penicillin and 50 ​μg/ml streptomycin and 10 ​μM retinoic acid, as previously described [22]. Seventy-two hours later, differentiated cells were subjected to transient transfection. To model conditions reflecting impaired biological processes in ALS, we exposed differentiated NSC-34 ​cells to the neurotoxic non-protein amino acid β-N-methylamino-l-alanine (L-BMAA; Merck, Milan, Italy). L-BMAA was originally isolated from the seeds of *Cycas circinalis* and later detected in the colonies of various species of cyanobacteria from marine, freshwater, and terrestrial environments, exposure to which was linked to the occurrence of neurodegenerative diseases, including ALS [23]. Forty-eight hours after transfection, cells were incubated with 500 ​μM L-BMAA for an additional 48 ​h.

### Assessment of cell survival determined as mitochondrial activity

Cytotoxicity was determined using the MTT assay, as previously reported [24]. In brief, the MTT solution was freshly prepared by dissolving 0.5 ​mg/ml in phosphate buffered saline (PBS), then sterilely filtered, and incubated for 1 ​h in the dark at 37 ​°C, 5 ​% CO_2_ in a humidified atmosphere. The insoluble purple formazan product was dissolved in 0,04 ​N acidic 2-propanol and its amount was determined by measuring the absorbance (Optical Density, OD) at 570 ​nm using a Biospectrometer® (Eppendorf, Hamburg, Germany). Background subtraction was measured at 650 ​nm. Data were expressed as a percentage of the absorbance measured in untreated cells (OD treated/OD untreated ​× ​100 ​%). Data were presented as the mean ​± ​S.E.M.

### Transient transfection

Differentiated NSC-34 ​cells were transiently transfected with preselected siRNAs against mouse ZnT1 using HiPerFect Transfection Reagent and according to the manufacturer's recommendations. After culturing for 72 ​h, a complex of ZnT1 siRNA (siZnT1)/HyPerFect or a negative control siRNA (siCTL) at 25 ​nmol/L was added to differentiation medium and kept at 37 ​°C and 5 ​% CO2 for an additional 5 ​h. At the end of the incubation period, fresh complete culture medium was added for an additional 24 ​h in a humidified incubator.

NSC-34 ​cells were transfected with 5 ​nM Mimic Let-7a Mimicor 1 ​nM specific anti-Let-7amiRNA inhibitor or the corresponding negative control (NT-miR) using HiPerFect Transfection Reagent according to the manufacturer's recommendations. Functional assays and protein analysis were performed 48 and 72 ​h later.

### Real-time PCR

Mice were deeply anesthetized with 3 ​% isoflurane vaporized in O_2_/N_2_O 50:50 and sacrificed. The spinal cord was rapidly removed and immediately frozen on dry ice and stored at −80 ​°C until use. Total RNA was extracted following supplier's instructions (Life Technologies) and cDNA was synthesized using 0.5 or 2g of total RNA to obtain miRNA specific cDNA or total cDNA, respectively, with the High Capacity Transcription Kit following supplier's instruction (Life Technologies, Monza, Italy). Quantitative real-time PCR was performed with TaqMan assays in a 7500 real-time PCR system (Life Technologies). Changes in miRNA and mRNA levels were determined as the difference in threshold cycle (2ˆ-ΔΔCt) between the target gene Slc30a1 (ID:Mm00437377_m1) or miRNA let-7a-5p (ID: 000377) and the appropriate reference gene: beta-glucuronidase (Gusb,ID:Mm00446953_m1)and U6 snRNA (ID:001973), as previously reported [25].

### Cloning of the SLC30a1 3′ UTR

PCR amplification was performed using PrimeSTAR® GLX DNA polymerase (Takara, Kusatsu, JP) on human cDNA, priming at +2451 bp respect to the stop codon to +3775 bp (GenBank accession number NM_021194.2), inserting an *Nhe*I and a *Xho*I restriction site within the forward and the reverse primers, respectively. The PCR product was purified using StrataPrep DNA Gel Extraction Kits (Agilent, Milan, Italy) and cloned into multiple cloning sites of pmirGLO Dual-Luciferase miRNA Target Expression Vector (Promega, Milan, Italy) downstream of the firefly luciferase gene (luc2). The primer sequences flanked by *Nhe*I and *Xho*I sites used for the amplification were: Znt1-3′UTR fwd:5′-atagctagcgagttgcatctctcctgctgg-3′ and Znt1-3′UTR rev:5′-gcgctcgagaagaaacacagtagtctgtctca-3’. The primer used for site-directed mutagenesis were: Znt-3′UTR mut fwd: 5′-gttattttccaaagctgttccggatccaccatgaggctttatggattg-3′ and Kif5A-3′UTR mut rev: 5′- caatccataaagcctcatggtggatccggaacagctttggaaaataac-3′that inserted a *Bam*HI restriction site, useful for colony screening. The fidelity of the constructs was confirmed by DNA sequencing [26].

### Dual-luciferase reporter gene assay

PC12 ​cell line was maintained in culture in complete RPMI 1640 medium (RPMI, 10 ​% heat-inactivated HS, 5 ​% FBS, 1 ​% l-Glutammine, 1 ​% Penicillin-Streptomycin) and plated 24 ​h before transfection in a 24-well plate at a concentration of 40000 ​cell/well. PC12 cells were transfected with 100 ​ng of each plasmid in combination with 30 ​nM of let-7a-5p mimics, (Mimic; Ambion, Milan, Italy) or specific miRNA inhibitors (anti-miR-Let7a; Ambion) or the negative control (NT-miR; Ambion) with Lipofectamine 2000 (Invitrogen) according to the manufacturer protocol. Forty-eight hours after transfection, the dual luciferase assay was performed following the supplier's instructions (Promega) and measured with a manual luminometer (Glomax 20/20, Promega). The effect of 3′-UTR activity on the reporter gene was calculated as a firefly-to-renilla ratio [26].

### Preparation of cellular and tissue protein extracts

After twice washing with ice-cold PBS, NSC34 ​cell cultures were harvested with a cell scraper and centrifuged at 6000×*g* for 10 ​min. After decanting PBS, the cell pellets were lysed with RIPA Buffer (150 ​mM NaCl, 50 ​mM Tris-HCl, 1 ​mM EDTA, 1 ​% NP-40, 0.05 ​% DOC, 0.1 ​% SDS, 1 ​mM Na_3_VO_4_, 1 ​mM NaF) supplemented with 1 ​mM PMSF and 1:50. Protease Inhibitor Cocktail (Merck Life Science, Milan, Italy). Total protein lysates of the tissue samples were immersed in ice-cold isolation buffer as reported [27]. In brief, spinal cord samples were homogenized using a Potter-Elvehjem PTFE pestle and a glass tube in a lysis buffer containing in mM: 250 sucrose, 10 KCl, 1.5 MgCl_2_, 1 EDTA, 1 EGTA, 1 dithiothreitol, 20 ​mM 4-(2-hydroxyethyl)-1-piperazineethanesulfonic acid (HEPES) pH 7.5, 0.2 ​% SDS and supplemented with 1 ​mM PMSF and 1:50 Protease Inhibitor Cocktail. At the end of the solubilization phase, the cell and tissue protein preparations were centrifuged at 16,000×*g* for 10 ​min, and the supernatants containing the total protein lysates were used to estimate the protein content using the Bio-Rad Protein Assay according to the Bradford method (Bio-Rad Laboratories, Milan, Italy) [28].

### Western blotting

Four concentrated denaturing buffer (250 ​mM Tris, pH 6.8, 5 ​% SDS, 40 ​% glycerol, 0.005 ​% bromophenol blue, and 10 ​% 2-mercaptoethanol) were used to denature the samples for 5 ​min at 95 ​°C and then resolved by SDS-PAGE using 8–10 ​% polyacrylamide gels. Following electrophoresis, proteins were electro transferred onto pre-wet with methanol 0.2 ​μm PVDF membranes (Bio-Rad Laboratories, Milan, Italy) and were immunoblotted O.N or for 1 ​h with: rabbit polyclonal anti-ZnT1 antibody (1:2000; SYSY GmbH, Göttingen, Germany) or mouse monoclonal α-Tubulin antibody (1:2000; Cell Signaling Technology, Inc., Danvers, MA, USA) or rabbit polyclonal β-actin antibody (1:2000; Cell Signaling Technology, Inc., Dancers, MA, USA). After being washed three times with Tris-buffered saline with Tween 20 (TBS-T) before and after incubation with HRP-conjugated secondary antibodies (1:2000 for 1 ​h at room temperature; Life Technologies, Italy), protein band was detected using enhanced chemiluminescence (ECL) HRP substrate for Western blotting (SuperSignal™ West Pico PLUS Chemiluminescent Substrate, Pierce, Life Technologies) and scanned on ChemiDoc Imaging System (Bio-Rad). Image processing and quantification of Western blot bands were performed using ImageJ software and normalized to a loading control protein (α-tubulin or β-actin) [20].

### Animal model

B6SJL-TgN SOD1/G93A(+)1Gur mice expressing a high copy number of mutant human SOD1 with a Gly93Ala substitution (SOD1^G93A^) and B6SJL-TgN (SOD1) 2Gur mice expressing wild-type human SOD1 (WT) were obtained from Jackson Laboratories (Bar Harbor, ME, USA). This model was used for experiments as it remains the only validated mouse model of ALS for preclinical study according to the ALS Therapy Development Institute and reproduces much of the pathogenesis and pathology of clinical disease. Transgenic animals have been crossed with background-matched B6SJL wild-type females, and selective breeding maintained each transgene in the homozygous state. All transgenic mice were identified analyzing DNA extracts from tail tips by PCR as previously described [29,30]. Overall, 80 male and female mice were group housed in microisolator caging under standard 12-h light dark conditions with access to food and water. 7 males and 5 females out of 80 animals were not included in the experimental groups as they died for unknown reasons. The number of female and male mice was balanced among all the experimental groups. Dead animals were equally distributed among the experimental groups. Experiments were performed according to the international guidelines for animal research and approved by the Animal Care Committee of “Federico II” University of Naples, Italy and Ministry of Health, Italy. All efforts were made to minimize animal suffering and to reduce the number of animals used.

### anti-Let7A-5p treatment in mice

Anti-Let7a-5p, diluted at the final concentration of 10 ​μM in a previously filtered saline solution (0.9 ​% NaCl g/L), was administered in the right lateral ventricle once a week for 5 weeks starting from day 90 of age at the volume of 2 ​μl. Intracerebroventricular administration was carried out in mice positioned on a stereotaxic frame through a 27 ​g stainless steel cannula implanted into the right lateral ventricle at the following coordinates from the bregma:+0.6 ​mm caudal, −1.6 ​mm lateral and 2.3 ​mm below the dura. The dose was chosen as previously described [20,31].

### Tissue processing, immunostaining, and confocal immunofluorescence

Spinal cords were rapidly removed on ice and postfixed overnight at +4 ​°C and cryoprotected in 30 ​% sucrose in 0.1 ​M phosphate buffer (PB) with sodium azide 0.02 ​% for 24 ​h at 4 ​°C. Spinal cords were then sectioned frozen on a sliding cryostat at 40 ​μm thickness, in rostrum-caudal direction. Afterward, free floating serial sections were incubated with PB Triton X 0.3 ​% and blocking solution (0.5 ​% milk, 10 ​% FBS, 1 ​% BSA) for 1 ​h and 30 ​min. The sections were incubated overnight at +4 ​°C with the following primary antibodies: anti-SMI32 (mouse monoclonal antibody; 1:1000; Biolegend, San Diego, CA), anti-GFAP (Rabbit polyclonal antibody 1:000: Millipore, Darmstadt, Germany), anti-Iba1 Rabbit polyclonal antibody; 1:1000; Wako, Japan), anti-ZnT1 (Rabbit polyclonal antibody 1:1000: AlomoneLab, Dallas, Texas.). The sections were then incubated with the corresponding florescent-labeled secondary antibodies, Alexa 488/Alexa 594 conjugated antimouse/antirabbit IgGs (Jackson ImmunoResearch Baltimore, PA). Nuclei were counterstained with Hoechst (Sigma-Aldrich, Milan, Italy). Images were observed using a Zeiss LSM700 META/laser scanning confocal microscope (Zeiss, Oberkochen, Germany). Single images were taken with an optical thickness of 0.7 ​μm and a resolution of 1024 ​× ​1024. In double-labeled sections, the pattern of immune reactivity for both antigens were identical to that seen in single-stained material. Tissue labeling without primary antibodies was also tested to exclude autofluorescence. No specific staining was observed under these control conditions, thus confirming the specificity of the immunosignals [32]. Nissl staining was performed as previously described [33]. Briefly, slide-mounted sections were dipped 7 ​min in 0.5 ​% solution of Cresyl Violet in distilled water supplemented with acetic acid (16 ​N solution, 60 drops/l). Slides were then rinsed in distilled water, dehydrated through graded ethanol baths (95 ​%, 100 ​%; 5 ​min each), delipidated 8 ​min in xylene, and coverslipped with Eukitt Mounting Medium (Bio-Optica, Milan, Italy).

#### miR let-7a-5p expression by fluorescence in situ hybridization (FISH)

For in situ hybridization, all procedures were performed in autoclaved solutions and RNAse-free conditions. mice were perfused with 1 ​× ​PBS and 4 ​% paraformaldehyde solution in PBS. Frozen tissue sections were prepared following the description of miRNA protocol for in situ hybridization on frozen sections (Exiqon).

Briefly, spinal cord sections were submerged in neutral buffered formalin (10 ​%) for 15 ​min and then washed in PBS three times for 5 ​min. Sections were incubated in proteinase K buffer containing 1 ​M Tris–HCl (pH 7.4), 0.5 ​M EDTA, 5 ​M NaCl, and proteinase K (15 ​μg/mL) in RNase-free water for 10 ​min at 37 ​°C, and then, the sections were washed three times for 3 ​min in PBS. Sections were then incubated in 3 ​% H2O2 for 5 ​min to inhibit endogenous peroxidase activity and then washed in PBS three times for 3 ​min. Sections were sequentially hybridized for 1,30 ​h at 55° for mir-Let-7a-5p (5′DIG and 3′DIG). The final concentration of the probe was 20 ​nM. The sections were hybridized in hybridization buffer containing 50 ​% deionized formamide, 0.3 ​M NaCl, 20 ​mM Tris–HCl (pH 8.0), 5 ​mM EDTA, 10 ​mM NaPO4 (pH 8.0), 10 ​% dextran sulfate, 1 ​× ​Denhardt's solution, 0.5 ​mg/mL yeast RNA, and probe. Post-hybridization washes were performed sequentially twice for 5 ​min at hybridization temperature in 5 ​× ​saline sodium citrate (SSC) buffer, three times for 5 ​min at hybridization temperature in 1 ​× ​SSC buffer, twice for 5 ​min at hybridization temperature in 0.2 ​× ​SSC, and once for 5 ​min at room temperature in 0.2 ​× ​SSC buffer. Following the stringent washing, the sections were incubated in blocking solution containing 2 ​% sheep serum and 1 ​% BSA in PBS with 0.1 ​% Tween 20 for 15 ​min at room temperature. Then, the sections were incubated for 60 ​min with anti-digoxigenin-peroxidase (POD), antigen-binding fragments (Fabs; Roche Diagnostics) diluted 1:400 in 1 ​% sheep serum, 1 ​% BSA, and PBS with 0.05 ​% Tween 20 ​× ​. 9 Then, the sections were washed in PBS three times for 5 ​min and incubated for 5 ​min in Cy2-conjugated tyramide (tyramide signal amplification [TSA] Plus Fluorescein kit, PerkinElmer) by diluting TSA stock solution 1:50 in 1 ​× ​amplification diluent. Finally, sections were incubated with Hoechst for 40 ​min and mounted onto slides using Fluoromount aqueous mounting medium (Sigma) and stored in a dark room.

#### Motor neurons counting analysis

MNs were counted in the spinal cord. Sections of each area were analyzed as previously described ^34^. Frozen spinal cord was sectioned on a sliding cryostat at 20 ​μm, in rostrum-caudal direction. Analyses were performed using image J software in Polygonal-shaped neurons larger than 150 ​μm^2^ with a well-defined cytoplasm, nucleus, and nucleolus for MNs counting. Quantification of MNs was determined by counting and averaging 3 sections selected at equally spaced intervals spanning L1–6 under 20 ​× ​magnification, n ​= ​4 mice for each genotype were analyzed. Cell counting analysis was determined as total MNs per field (mm2) of 4.5 month-old mice.

#### Fluorescence intensity analysis

Quantification of GFAP, Iba1 fluorescence intensity on tissue sections at the level of the lumbar spinal cord (L1-L6), was quantified in terms of pixel intensity value by using the NIH image software, as previously described [34]. Briefly, digital images were taken with ​× ​20 or ​× ​10 objective and identical laser power settings and exposure times were applied to all the photographs from each experimental set. Images were first thresholded to identify the positive signal; subsequently, the pixels expressing GFAP, and Iba1 were identified. Finally, the number of pixels positive for GFAP and Iba1 was measured per microscope field. Images from the same areas of each brain region were compared. Results were expressed in arbitrary units. n ​= ​3 mice per treatment group and 3 sections for each genotype.

#### ZnT1 cell counting analysis

Frozen spinal cord was sectioned on a sliding cryostat at 20 ​μm, in rostrum-caudal direction. Images from the same areas of each spinal cord region were compared. Analyses were performed using image J software in the total number of positive signals of ZnT1 for photographic field (mm2) in lumbar spinal cord (L1-L6) of WT and SOD1^G93A^ mice. n ​= ​3 mice per treatment group [35].

#### Evaluation of motor performance

The hind limb grip test was conducted by placing the mouse on a grid (45 ​cm long ​× ​28 ​cm large) upside-down (30 ​cm above a foam pad). The test was performed once a week and the latency to fall off the grid was also measured up to a maximum of 60 ​s. Paralysis onset was defined as the day in which the grip test performance was less than 20 ​% compared to day 1 performance. Motor coordination and balance were assessed using a five-station mouse rotarod apparatus (Ugo Basile; Milan, Italy) as previously described [36,37]. In each station, the rod was 6 ​cm in length and 3 ​cm in diameter. Mice were trained to maintain balance at increasing speed up to a constant speed of 14 ​rpm for three consecutive trials. The test sessions were conducted by one rotarod trial administered once a week. In this session, the speed of rotation was increased from 4 to 14 ​rpm over 180 ​s. Mice had three trials on the rod, and the latencies to fall were measured once a week and then averaged. The maximum latency of 180 ​s was assigned to mice that did not fall at all. Weekly evaluation of hind limb paralysis was performed. Hind limb paralysis was scored when the animal dragged one of its hind limbs, and paralysis of a forelimb was scored when the mouse failed to use its forelimbs for walking or righting. Body weight was measured immediately before each session of behavioral tests. The disease end stage was defined by the inability of mice to right themselves within 20 ​s when placed on their sides. We did not identify any differences between males and females for this reason we decided to use both genders.

#### Statistical analysis

Statistical analyses were performed using the software package GraphPad Prism v.8.0.2. (GraphPad Software, Inc., San Diego, CA). Data were expressed as mean ​± ​S.E.M. of the number of experiments indicated and performed in independent experimental sessions (at least three independent experimental sessions). Statistical significance was assessed by one-way analysis of variance (ANOVA) followed by Bonferroni's or Newman–Keuls method Multiple Comparison tests or Student's t-test for comparison between two experimental groups. Values were considered statistically significant when *p* value ​< ​0.05.

## Results

### ZnT1 expression was increased in the spinal cords of SOD1^G93A^ mice during the pre-symptomatic stage

**Fig. 1:**
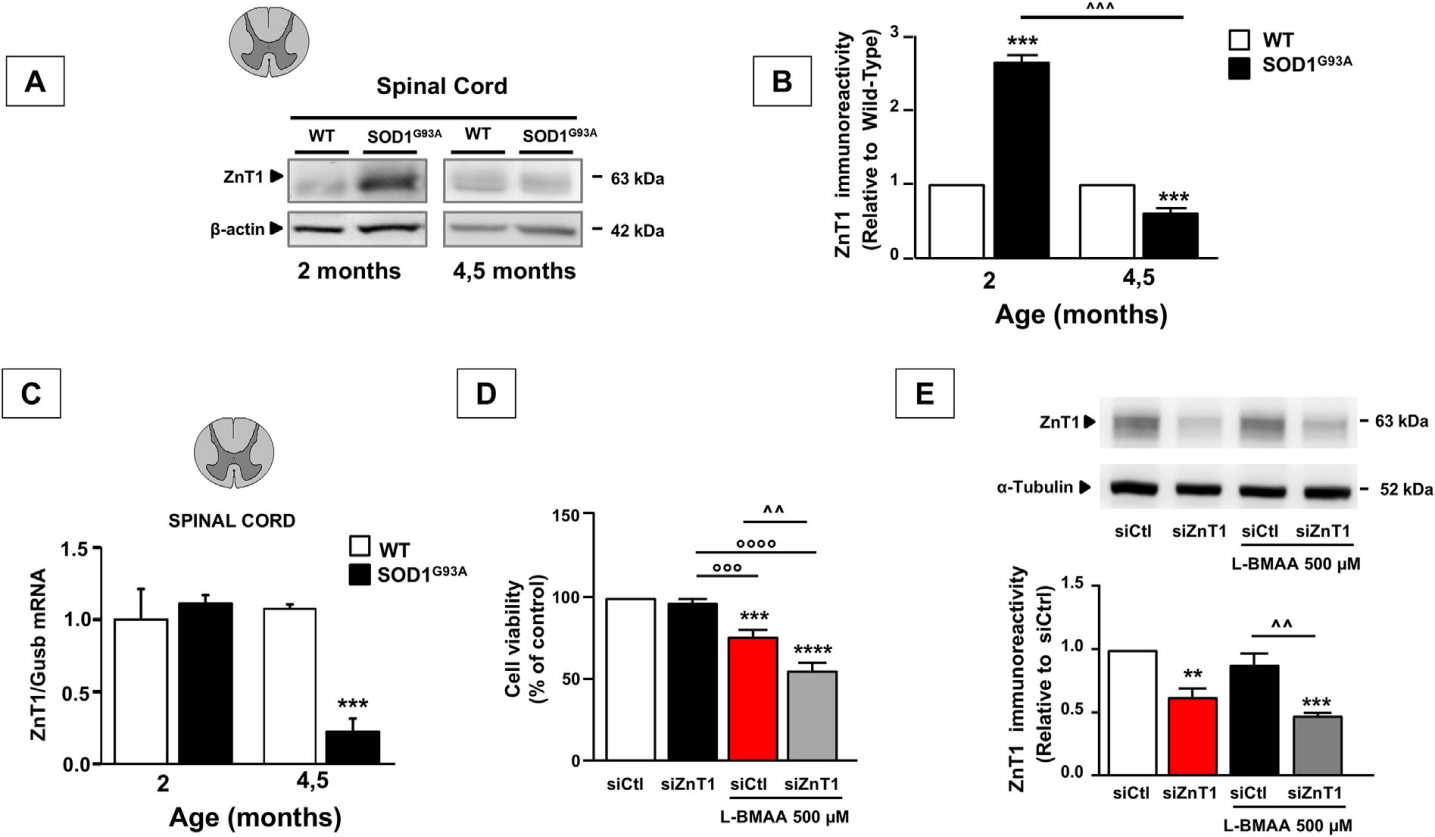
**Expression pattern of ZnT1 in the spinal cord of presymptomatic and symptomatic maleSOD1^G93A^ mice and effects of ZnT1 silencing on the viability of motor neurons exposed to the neurotoxin L-BMAA.** Representative Western blot (A) and densitometry quantification (B) of ZnT1 protein expression in the spinal cord of WT and SOD1^G93A^ male mice. (C) RT–PCR of ZnT1 mRNA expression in the spinal cord of WT and SOD1^G93A^ male mice. Values are presented as mean ​± ​SEM compared to the WT. Statistical significance was determined by One Way ANOVA followed by Newman-Keuls multiple comparison test with a confidence interval of 99 ​% (∗∗∗p ​< ​0.001 vs Wild-type; ˆ ˆ ˆ p ​< ​0,0001 10 weeks old SOD1^G93A^ vs 20 weeks old SOD1^G93A^). (D) Bar graph showing the viability of NSC-34 ​cells exposed to L-BMAA for 48 ​h and previously transfected with siControl or siZnT1. (E) Representative Western blot and corresponding densitometry quantification of ZnT1 protein expression in NSC-34 ​cells transfected with siControl or siZnT1 and exposed or not to L-BMAA. Statistical significance was determined by One Way ANOVA followed by Bonferroni multiple comparison test with a confidence interval of 95 ​% (∗∗p ​< ​0.01, ∗∗∗p ​< ​0.001, ∗∗∗∗p ​< ​0.0001 vs siCtl; °°°p ​< ​0.001, °°°° p ​< ​0.0001 vs siZnT1; ˆ ˆ p ​< ​0.01 vs siCtl ​+ ​L-BMAA).

To explore the role of ZnT1 in ALS progression, we evaluated its expression in spinal cord of presymptomatic (2-month-old) and symptomatic (4.5-month-old) SOD1^G93A^ mice. ZnT1 was strongly upregulated at 2 months of age (p ​< ​0.000001), whereas it was significantly less expressed at 4.5 months of age (p ​= ​0.000184) (Fig. 1A and B). To test whether the downregulation of ZnT1 protein observed in the symptomatic SOD1^G93A^ mice was paralleled by a reduction in transcription, we also examined the relative abundance of mRNA for ZnT1. In real-time PCR experiments, we found that ZnT1 mRNA levels did not differ between SOD1^G93A^ and WT mice at 2 months of age, whereas symptomatic SOD1^G93A^ mice showed lower mRNA levels for ZnT1 compared to age-matched WT mice (Fig. 1C).

### ZnT1 silencing worsened L-BMAA-induced motor neuronal cell death

To better characterize the contribution of ZnT1 in ALS, we evaluated the effects of silencing ZnT1 in differentiated NSC-34 ​cells exposed to the neurotoxin L-BMAA. The ZnT1 immunosignal was efficiently reduced in NSC-34 ​cells transfected with siRNA against the transporter (Fig. 1D). Interestingly, while the exposure to 500 ​μM L-BMAA for 48 ​h did not affect ZnT1 expression (Fig. 1D), when ZnT1 was knocked down, motor neuronal cell death induced by L-BMAA was significantly worsened (Fig. 1E).

### Let-7a-5p microRNA directly targeted ZnT1 and inversely correlated with ZnT1 protein expression in the spinal cord of SOD1^G93A^ mice

**Fig. 2:**
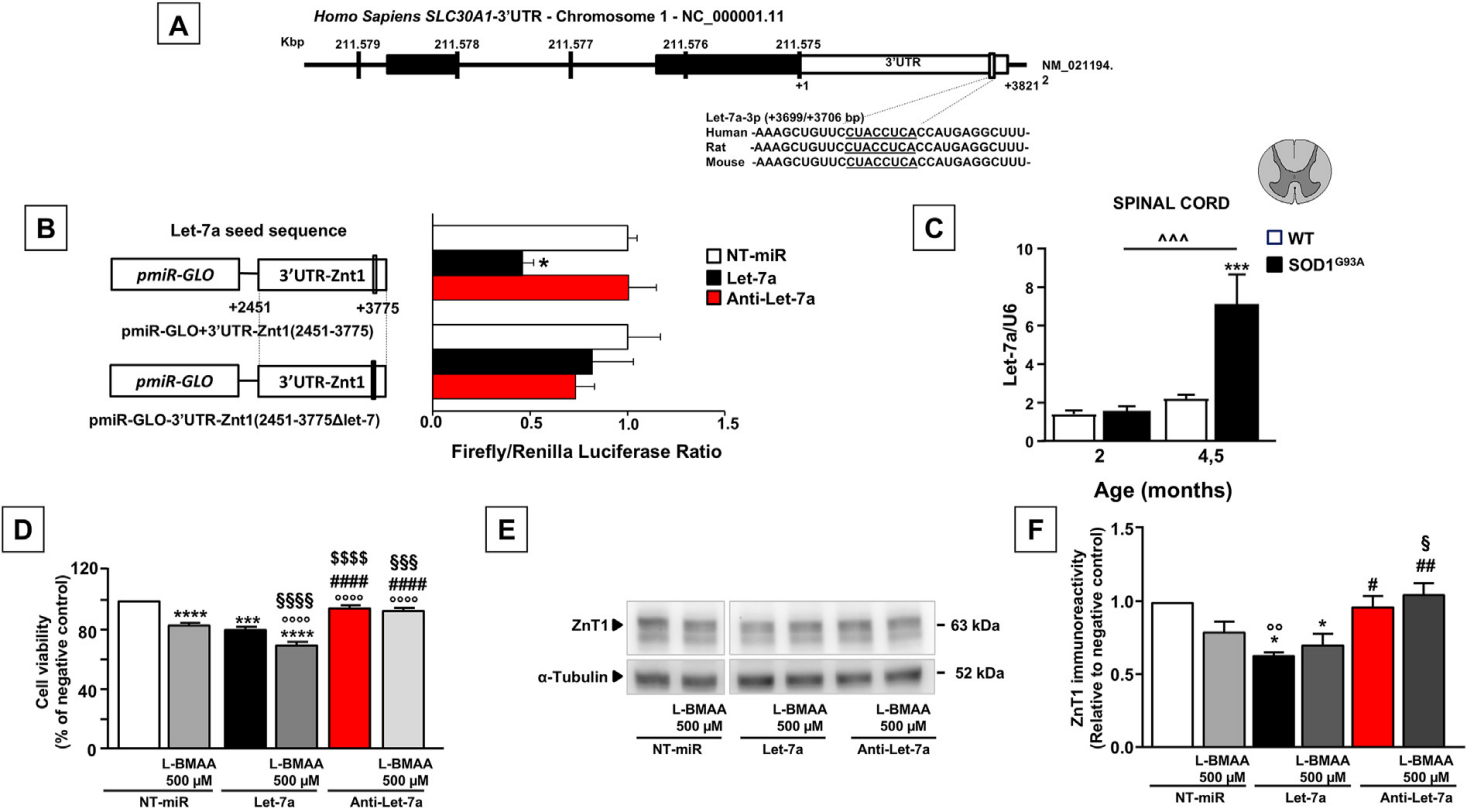
**Let-7a miRNA can bind ZnT1 mRNA predicted region and down-regulate ZnT1 transcript and protein levels, affecting motor neuron viability.** (A) Let-7a miRNA targeting to ZnT1 was predicted by miRNA target prediction databases. The sequences of seeds referring to the nucleotides in miRNA positions are shown. Watson–Crick matches in the seed sequence are highlighted. (B) Luciferase assay was performed in PC12 ​cells transfected with Let-7a and the pmirGLO Luciferase Expression Vector containing the wild type 3′UTR of ZnT1 gene or the 3′UTR-ZnT1 mutated on the Let-7a seed sequence. (C) RT–PCR of Let-7a expression in the spinal cord of WT and SOD1^G93A^ male mice. Values are presented as mean ​± ​SEM compared to the WT. Statistical significance was determined by One Way ANOVA followed by Newman-Keuls multiple comparison test with a confidence interval of 95 ​% (∗∗∗p ​< ​0.001 vs 4.5 months old WT; ˆ ˆ ˆ p ​< ​0.001 vs 2 months old WT). (D) Measurement of cell viability in NSC-34 ​cells prior transfected with Let-7a mimic or the NT-miR or anti-Let-7a and then exposed or not to L-BMAA. (E) Representative Western blot and corresponding densitometry quantification of ZnT1 protein expression in NSC-34 ​cells transfected with Let-7a mimic or the NT-miR or anti-Let-7a and exposed or not to L-BMAA. Statistical significance was determined by One Way ANOVA followed by Bonferroni multiple comparison test with a confidence interval of 95 ​% (∗∗p ​< ​0.01, ∗∗∗∗p ​< ​0.0001 vs NT-miR; ###p ​< ​0.001 vs Let-7a; §§p ​< ​0.01 vs Let-7a ​+ ​L-BMAA).

With the aim of investigating the mechanisms underlying the down-regulation of ZnT1 mRNA and protein observed in the spinal cord of SOD1^G93A^ mice, we interrogated computational miRNA target prediction tools and predicted a putative miRNA binding site in the 3′ UTR target of the corresponding ZnT1 mRNA. Interestingly, a putative and highly conserved consensus sequence for Let-7a was identified in the 3′ UTR of the Slc30a1 gene encoding ZnT1 (Fig. 2A). A dual-luciferase reporter gene assay was then designed to verify the direct binding of Let-7a to the corresponding site of ZnT1 mRNA *in vitro*. Wild-type or mutant forms of the 3′ UTR of the miRNA expected to be the mRNA target were cloned downstream of a firefly luciferase reporter and co-transfected with Let-7a mimic or anti-Let-7a inhibitor in PC12 ​cells. Interestingly, Let-7a significantly (∼50 ​%) decreased luciferase signal intensity after transfection of the wild-type miRNA binding site analyzed in relation to the negative control group (P ​< ​0.05) (Fig. 2B). In contrast, transfection of the complementary sequence anti-Let-7a showed no noticeable change in luciferase signal intensity. Most importantly, targeted mutation of the putative Let-7a binding site completely abolished the Let-7a-induced decrease in reporter gene expression (Fig. 2B). This suggests that Let-7a can directly target and negatively regulate ZnT1 by binding its 3ʹ-UTR contributing to the overall post-transcriptional regulation of the gene. Having shown that Let-7a is a direct regulator of ZnT1 protein expression, we next analyzed Let-7a expression levels in the spinal cord of 2- and 4.5-month-old SOD1^G93A^ and WT littermates. Interestingly, we found that Let-7a was overexpressed in the spinal cord samples of SOD1^G93A^ mice at the end of the disease (4.5 months) compared to age-matched WT mice (Fig. 2C). In contrast, there were no significant changes in Let-7a expression between 2-month-old SOD1^G93A^ and WT mice (Fig. 2C), suggesting that the upregulation of Let-7a may contribute to the ZnT1 reduction observed in the late phase of the disease.

### anti-Let-7a-5p inhibitor rescued L-BMAA-induced motor neuronal cell death and prevented ZnT1 down-regulation

To characterize the Let-7a-dependent ZnT1 regulation in ALS, Let-7a expression was modified by transfection of Let-7a mimic or anti-Let-7a in differentiated NSC-34 ​cells exposed to L-BMAA, an *in vitro* model of ALS. Differentiated NSC-34 ​cells were previously transfected with Let-7a mimic or anti-Let-7a inhibitor and then exposed to L-BMAA for 48 ​h. As shown in Fig. 2D, ectopic expression of Let-7a significantly reduced motor neuron viability compared to transfected negative control cells. In contrast, in cells transfected with synthetic anti-Let-7a inhibitor there were no significant changes in cell viability (Fig. 2D). Intriguingly, ectopic overexpression of Let-7a exacerbated L-BMAA-induced cytotoxicity (Fig. 2D) and resulted in a further reduction in cell viability compared to cells exposed to L-BMAA and Let-7a alone. Most importantly, overexpression of synthetic anti-Let-7a inhibitor completely prevented the cytotoxic effect of L-BMAA (Fig. 2D). At the same time, we investigated whether the expression of ZnT1 was altered by the forced expression of Let-7a under these experimental conditions. As shown in Fig. 2E and F and in agreement with previous data obtained in PC12 ​cells, ectopic expression of Let-7a mimic reduced ZnT1 protein content. In contrast, no significant changes in ZnT1 protein levels were detected after transfection with the synthetic anti-Let-7a inhibitor compared to the negative control (Fig. 2E and F). In addition, upon chronic exposure to L-BMAA, a slight but non-significant decrease in ZnT1 protein expression occurred (p ​= ​0.4611). Prior upregulation of Let-7a contributed to a reduction in ZnT1 protein levels (∗∗p ​< ​0.001 compared to the negative control), while transfection with an anti-Let-7a inhibitor partially prevented ZnT1 downregulation (Fig. 2E and F), likely lowering endogenous Let-7a levels. This suggests that the shift toward greater toxicity of L-BMAA in Let-7a overexpressing NSC-34 ​cells appears to be dependent on significant downregulation of ZnT1 (Fig. 2D).

Consistent with this hypothesis, preventing ZnT1 downregulation by transfection of anti-Let-7a had a protective effect upon chronic L-BMAA exposure (Fig. 2D).

### anti-Let7a-5p treatment decreased Let7a-5p miRNA expression in motor neurons of SOD1^G93A^ mice

**Fig. 3:**
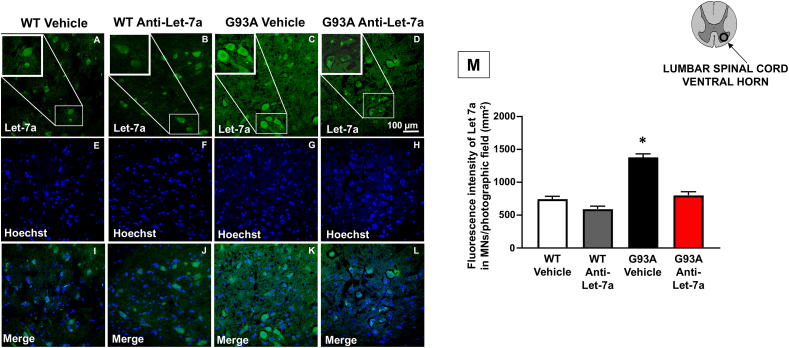
**Let-7a immunolocalization and quantification in spinal cord ofSOD1^G93A^mice treated to Anti-Let-7a.** Double labeling of Let-7a(green) and Hoechst (blue) and Merge (yellow) in spinal cord of WT mice ​+ ​vehicle (a,e,i), WT mice ​+ ​anti-Let-7a (b,f,j) G93A mice ​+ ​vehicle (c,g,h) SOD1^G93A^ mice ​+ ​anti-Let-7a (d,h,l) Scale bar 25 ​μm. Q: Fluorescence intensity in WT, SOD1^G93A^ treated with vehicle and WT, SOD1^G93A^ treated with anti-Let-7a. Data are expressed as mean ​± ​SEM (n ​= ​3 for each group). ∗p ​< ​0.05, SOD1^G93A^ anti-Let-7a vs. SOD1^G93A^ Vehicle. P values were obtained one-way ANOVA with Newman Keuls's correction for multiple comparisons.

To verify if the in vivo treatment with anti-Let-7a effectively modified the expression of the miRNA, in situ fluorescence hybridization experiments were conducted. As shown in Fig. 3, the microRNA Let-7a was primarily expressed in larger neurons with a triangular shape, identified as the motor neurons of the ventral spinal cord. The expression of Let-7a was both cytoplasmic and nuclear, as indicated by the colocalization with the nuclear marker, Hoechst, in all experimental groups (Fig. 3I-L). Notably, the expression was higher in the SOD1^G93A^ group treated with the vehicle, and the fluorescence intensity signal significantly decreased in the SOD1^G93A^ group treated with anti-Let-7a, indicating that the treatment could inhibit miRNA Let-7a expression (Fig. 3C and D). Additionally, fluorescence intensity calculated specifically in neurons larger than 15 ​μm confirmed the reduction of the signal in the SOD1^G93A^ group treated with anti-Let-7a matched to the vehicle-treated group (Fig. 3M).

### anti-Let7a-5p treatment increased ZnT1 expression in motor neurons of SOD1^G93A^ mice

**Fig. 4:**
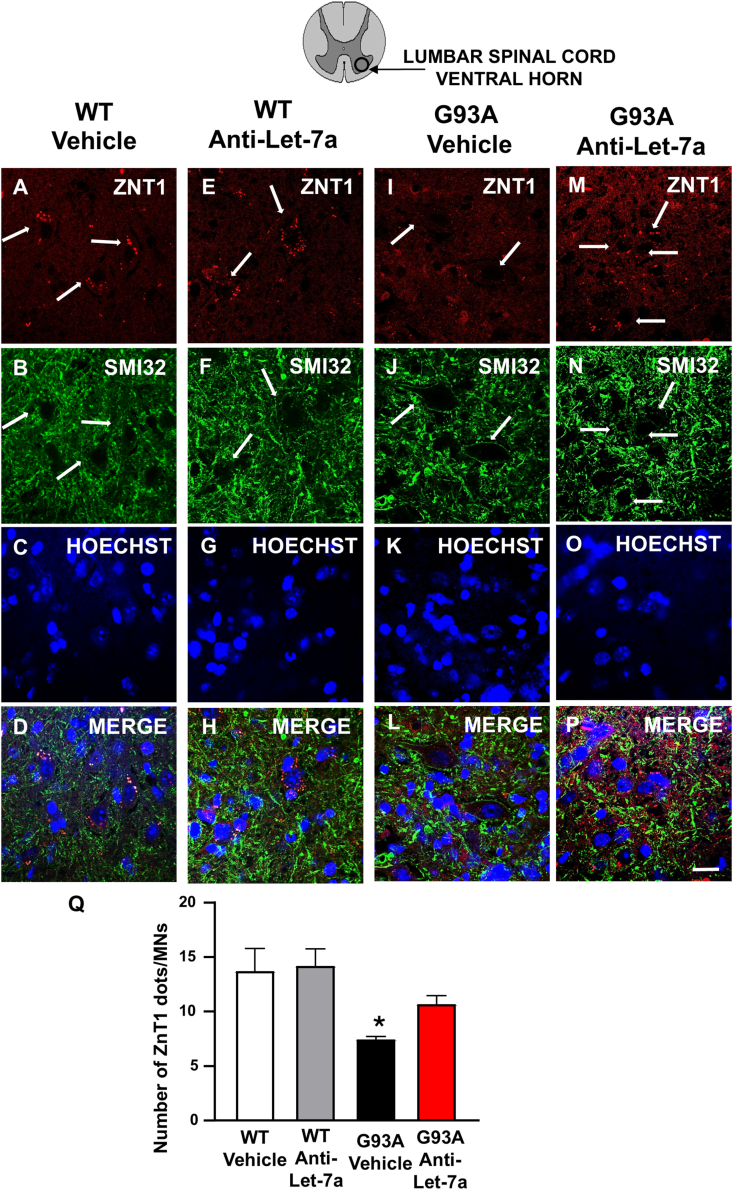
**Zn**T**1 immunolocalization and quantification in the spinal cord ofSOD1^G93A^mice treated to Anti-Let-7a.** Double labeling of ZnT1(red) and SMI32 (green), Hoechst (blue) and Merge (yellow) in spinal cord of WT mice ​+ ​vehicle (a–d), WT mice ​+ ​anti-Let7a (i–l) G93A mice ​+ ​vehicle (e–h) G93A mice ​+ ​anti-Let7a (m–p) Scale bar 25 ​μm. Q: Number of ZnT1 dots in WT, G93A treated with vehicle and WT , G93A treated with anti-Let7a. Data are expressed as mean ​± ​SEM (n ​= ​3 for each group). ∗p ​< ​0.05, G93A anti-Let7a vs. G93A Vehicle. P values were obtained one-way ANOVA with Newman Keuls's correction for multiple comparisons.

Confocal microscopy experiments were performed to evaluate whether treatment with anti-Let7a increased the expression of ZnT1 in motor neurons and to elucidate the localization of ZnT1. As shown in Fig. 4, ZnT1 expression was detected as a punctuate staining in the cytosol of motor neurons labeled with the SMI32 marker in WT mice (Fig. 4A–D). The punctate ZnT1 signal was reduced in motor neurons of SOD1^G93A^ vehicle mice (Fig. 4E) and treatment with anti-Let7a was able to restore ZnT1 signaling in motor neurons (Fig. 4M ​− ​P, Q).

Indeed, as shown in the graph, a significant decrease in the number of ZnT1 dots in motor neurons of G93A animals treated with the vehicle compared to the wild-type group was observed. Conversely, treatment with anti-Let7a induced a significant increase in the number of ZnT1 dots in SOD1^G93A^ mice (Fig. 4Q).

### Prolonged anti-Let7a-5p treatment delayed disease onset and increased survival rate in SOD1^G93A^ mice

To assess whether anti-Let7a could improve survival and motor symptoms, anti-Let7a and the vehicle were intracerebroventricularly infused in SOD1^G93A^ mice, starting from the two months of age, the presymptomatic phase. Administration was carried out once a week for 5 weeks, and motor tests were carried out once a week. At the end of the 5th week, the animals were sacrificed for histological and biochemical investigations. We did not observe any effect of anti-Let 7a on motor behavior, body weight, or survival in wild-type animals. Therefore, the corresponding results have not been reported.

**Fig. 5:**
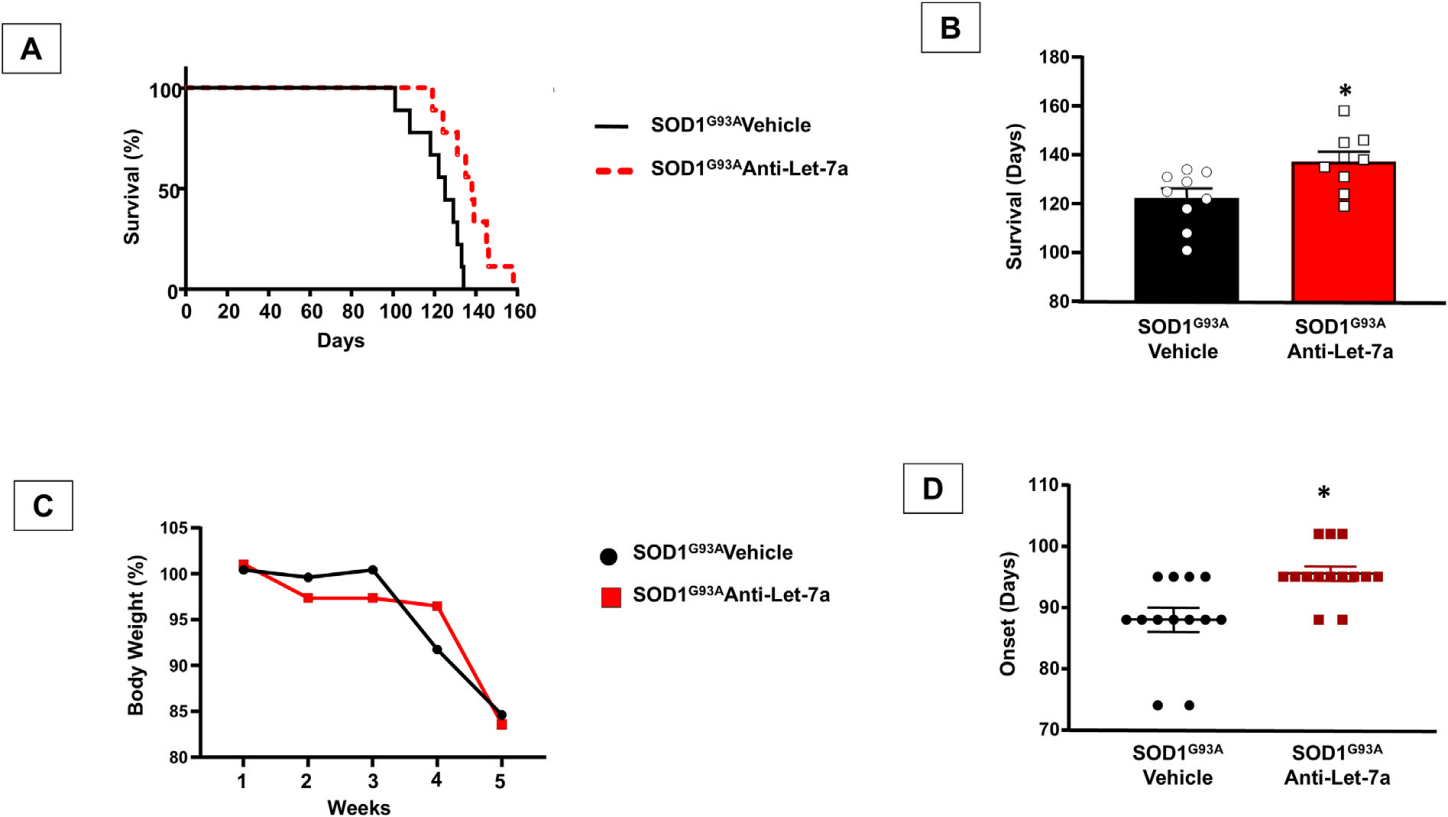
**Effect of Antilet-7a on survival, onset of disease and body weight ofSOD1^G93A^mice.** Kaplan-Meier survival analysis (A), expressed as a percentage and in days (B), n ​= ​9 SOD1^G93A^ mice treated with vehicle (white circle) and n ​= ​9 SOD1^G93A^ mice treated with anti-Let7a (white square). Statistical analysis was performed using a Student's *t*-Test; significant differences are indicated as: ∗p ​< ​0.05, SOD1^G93A^ mice anti-Let7a vs. SOD1^G93A^ mice Vehicle. (C): Body weight expressed in percentage of SOD1^G93A^ mice treated with vehicle (n ​= ​13) and SOD1^G93A^ mice treated with anti-Let7a (n ​= ​14). P values were obtained using one-way ANOVA with Newman Keuls's correction for multiple comparisons. (D): Paralysis onset analysis expressed in days of G93A mice treated with vehicle (n ​= ​13) and G93A mice treated with anti-Let7a (n ​= ​14). Statistical analysis was performed using a Student's *t*-Test; significant differences are indicated as: ∗p ​< ​0.05.

As concern survival rate, anti-Let7a prolonged the lifespan of ALS mice. In fact, the mean lifespan of SOD1^G93A^ mice treated with vehicles was 122.3 ​± ​3.8 days, while that of ALS mice treated with anti-Let7a was significantly markedly longer, being 137.2 ​± ​3.9 days (Fig. 5A and B).

Regarding body weight, SOD1^G93A^ mice treated with the vehicle showed weight loss starting at 4 weeks post-treatment, with a notable 10 ​% decrease in body weight recorded. By contrast, body weight remained constant in G93A mice treated with anti-Let7a for 4 weeks (Fig. 5C).

Regarding the onset of the disease, it was observed that muscle atrophy due to denervation resulted in a 15–20 ​% decrease in motor performance, as assessed by grip strength tests and the rotarod. The paralysis onset in SOD1^G93A^ mice treated with the vehicle was 88 ​± ​2.1 days, while in SOD1^G93A^ mice treated with anti-Let7a, it was 95.5 ​± ​1.5 days. (Fig. 5D).

### anti-Let7a-5p treatment prolonged motor neuron survival and ameliorates motor symptoms in SOD1^G93A^ mice

**Fig. 6:**
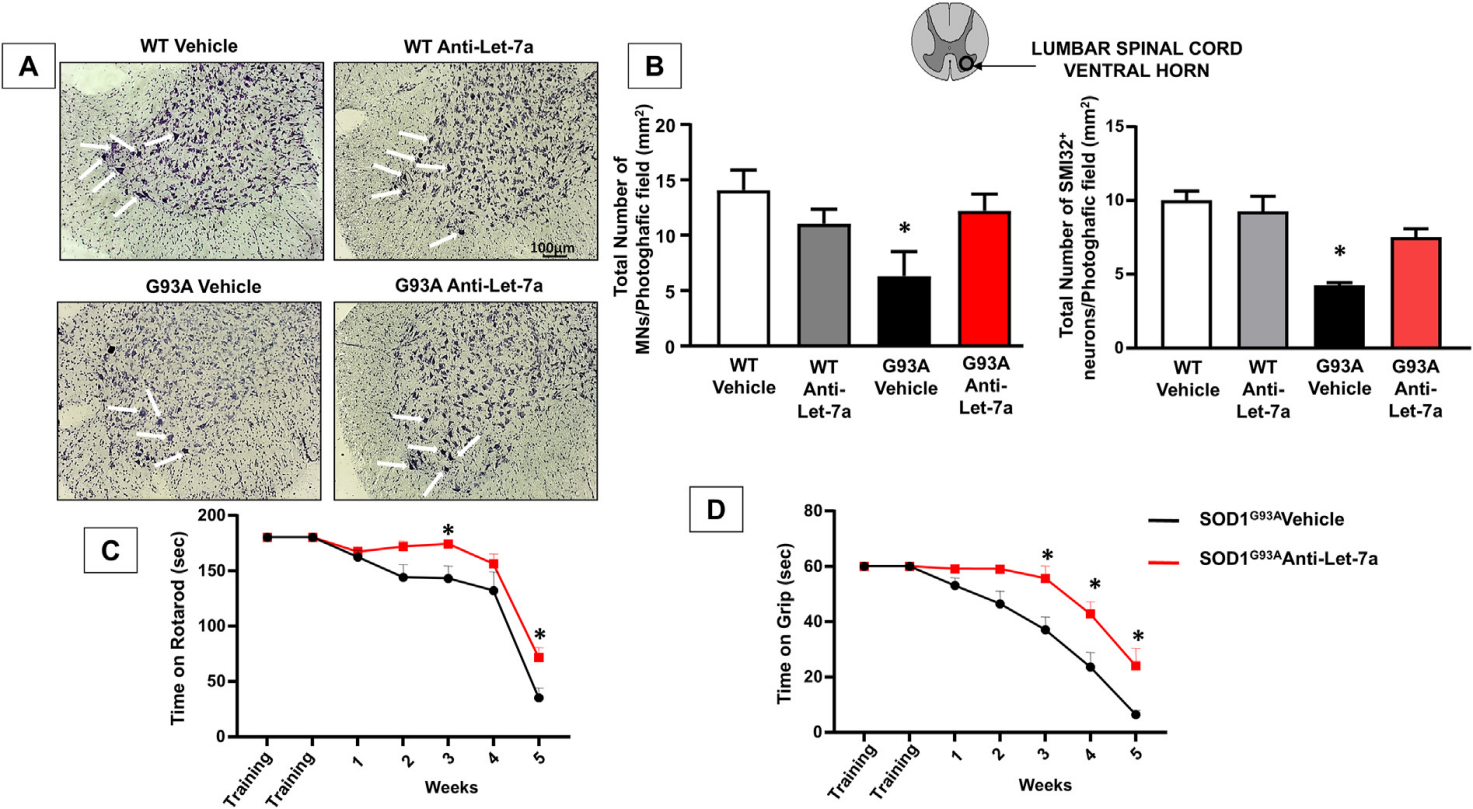
**Effect of Antilet-7a on motor neuron survival and motor functions ofSOD1^G93A^mice.** (A): Representative image of Nissl staining in the spinal cord. Scale bar 100 ​μm. (B): Cell counting analysis of motor neurons expressed as a total number of motor neurons and SMI32 positive cells per photographic field in the spinal cord of SOD1^G93A^ mice treated with vehicle or anti-Let7a compared to wild-type vehicle and anti-Let7a. ∗p ​< ​0.05, SOD1^G93A^ mice vehicle vs SOD1^G93A^ mice anti-Let7a. Data are expressed as mean ​± ​SEM (n ​= ​3/4 for each group). P values were obtained using one-way ANOVA with Newman Keuls's correction for multiple comparisons. (C): Rotarod test expressed in seconds in SOD1^G93A^ mice treated with vehicle (n ​= ​13) or anti-Let7a (n ​= ​14). P values were obtained using one-way ANOVA with Newman Keuls's correction for multiple comparisons. Significant differences are indicated as: ∗p ​< ​0.05. (D) Grip performance expressed in seconds in SOD1^G93A^ mice treated with vehicle (n ​= ​13) or anti-Let7a (n ​= ​14). P values were obtained using one-way ANOVA with Newman Keuls's correction for multiple comparisons. Significant differences are indicated as: ∗p ​< ​0.05.

To determine if anti-Let7a treatment had a neuroprotective effect on motor neurons, spinal cord sections from fully symptomatic mice were stained using Nissl staining, and the number of MNs was counted in the ventral region of the spinal cord (Fig. 6A). Given that degeneration in ALS pathophysiology predominantly affects large motor neurons, the count of cells with a perikaryal projection area exceeding 150 ​μm^2^ was conducted in wild-type and fully symptomatic SOD1^G93A^ mice that were chronically treated with either a vehicle or anti-Let7a. The results indicated that anti-Let7a treatment protected large motor neurons in the spinal cords of fully symptomatic SOD1^G93A^ mice. Mice treated with anti-Let7a exhibited a greater number of spinal MNs (12 ​± ​1.5) compared to those treated with the vehicle (6.3 ​± ​2.2) (Fig. 6B). The neuroprotective effect of anti-Let7a on motor neurons was verified using the SMI32 antibody, a specific marker for MNs. The total number of SMI32-positive cells was indeed higher in the anti-Let7a SOD1^G93A^ mice group compared to the vehicle group. (Fig. 6B).

To determine if the reduced loss of MNs in anti-Let7a-treated SOD1^G93A^ mice was associated with improved motor performance, behavioral tests were conducted weekly on wild-type and SOD1^G93A^ mice. These tests began in the second month after birth (P60), coinciding with the start of ICV administration of anti-Let7a and when the mice were in the early symptomatic stage (Fig. 6C). The performance of SOD1^G93A^ mice treated with the vehicle deteriorated from slight impairment at 2 weeks of treatment to a complete loss of balance ability by 5 weeks of treatment, where the mice could only maintain balance for an average of 28 ​± ​seconds. In contrast, the performance of SOD1^G93A^ mice receiving anti-Let7a treatment declined at a slower rate, as indicated by significantly longer durations compared to those treated with the vehicle at week 5. Specifically, at 5 weeks, the anti-Let7a-treated SOD1^G93A^ mice were able to maintain their balance on the rotarod for an average of 71 ​s. The hindlimb grip test was conducted by placing the mouse on an upside-down grid, and the time taken for the mouse to fall off the grid was recorded, with a maximum measurement of 60 ​s (Fig. 6D). In the SOD1^G93A^ mice treated with the vehicle, the loss of limb strength became apparent after 3 weeks of treatment, with a recorded time of 39 ​± ​4.5 ​s. In fact, the grip strength on the grid decreased by approximately 50 ​% compared to the wild-type group, and at 5 weeks, the SOD1^G93A^ mice stayed on the grid for an average of just 7 ​s.

In ALS mice treated with anti-Let7a, grip performance was recorded at 55 ​s at 3 weeks, and by 5 weeks, the mice remained on the grid for an additional 24 ​s (Fig. 6D)

### anti-Let7a-5p treatment reduced astroglia and microglia activation in the spinal cord of SOD1^G93A^ mice

**Fig. 7:**
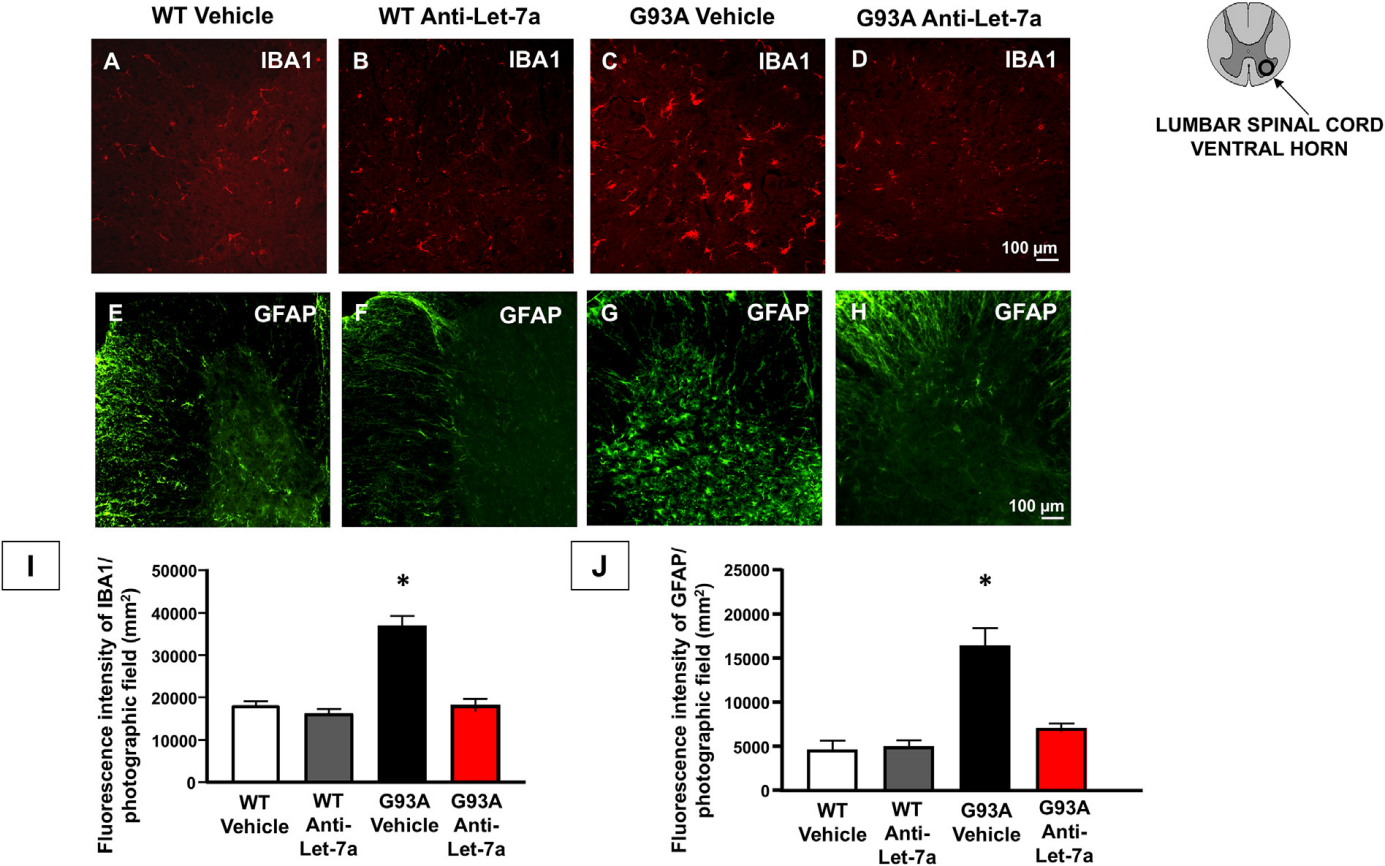
**Effect of Antilet-7a on glia activation ofSOD1^G93A^mice.** Immunofluorescence analysis of GFAP (E–H) and Iba1 (A–D) in spinal cord sections from vehicle or anti-Let7a of WTand SOD1^G93A^ mice (I,J) Fluorescence intensity of GFAP and Iba1 cells per photographic field (mm2), arbitrary units (AU). Data are expressed as mean ​± ​SEM (n ​= ​3/4 for each group). ∗p ​< ​0.05. P values were obtained using one-way ANOVA with Newman Keuls's correction for multiple comparisons.

To assess the effect of anti-Let7a treatment on astrogliosis and microgliosis, GFAP and Iba1 immunostaining were analyzed in the spinal cords of wild-type and SOD1^G93A^ mice treated with anti-Let7a. Scattered astrocytes were visualized through GFAP immunostaining in wild-type mice (Fig. 7E), and anti-Let7a treatment did not affect GFAP expression in these mice (Fig. 7F). In contrast, a pronounced astroglial reaction was observed in the vehicle-treated SOD1^G93A^ group (Fig. 7G), which was diminished in the SOD1^G93A^ mice treated with anti-Let7a (Fig. 7H). Indeed, the fluorescence intensity of GFAP labeling was reduced in SOD1^G93A^ mice treated chronically with anti-Let7a (Fig. 7J). Regarding microglia, a marked activation of Iba1 immunostaining was observed in SOD1^G93A^ mice treated with the vehicle (Fig. 7C). This activation was diminished in the mice treated with anti-Let7a (Fig. 7D). In fact, the fluorescence intensity of Iba1 labeling was significantly lower in SOD1^G93A^ mice chronically treated with anti-Let7a compared to those treated with the vehicle (Fig. 7I).

## Discussion

The present work demonstrated for the first time that in ALS SOD1^G93A^ mice, a reduction in ZnT1 expression consistent with disease progression occurs. Remarkably, through the use of a sequence complementary to the microRNA let-7a (anti-Let7a) able to modulate ZnT1 expression, we showed in ALS mice its capability to: (1) prevent the reduction in ZnT1 levels in the spinal cord; (2) preserve motor neuron survival in the ventral spinal horn; (3) decrease astroglial and microglial activation while sparing resident microglial cells in the spinal cord; and (4) improve the lifespan and alleviate motor symptoms.

Specifically, we identified miRNA Let-7a as an intriguing and novel target for post-transcriptional regulation of ZnT1 and demonstrated that Let-7a directly inhibits ZnT1 expression. Competitive inhibition of Let-7a by anti-Let-7a anti-miR effectively interfered with the Let-7a/ZnT1 axis and preserved ZnT1 levels and motor neurons *in vitro* and in vivo in ALS models. An imbalance in cellular zinc homeostasis can play a role in ALS [38]. Indeed, in the CSF of ALS patients elevated zinc concentration occurred [39,40] and an accumulation of labile zinc was observed in degenerating motor neurons and astrocytes of the spinal cord of animal model of the disease (SOD1^G93A^ mice), which can be effectively counteracted by intracellular zinc. The role of zinc in ALS has been described in different ALS models and several mechanisms have been identified as possible causes of the neurotoxic alterations in zinc concentration in ALS, including higher expression of zinc-permeable calcium-permeable AMPA/kainate channels (CAK) on motor neurons [41,42], reduced levels of zinc-binding proteins metallothionein I/II and III [43], decreased zinc-binding capacity of oxidized MTs upon oxidative stress [44], down-regulated expression of ZnT3 and ZnT6 zinc transporters [17]. On the other hand, the zinc transporter ZnT1 is crucial for lowering cytosolic zinc [45] following Zn loading, however its role in ALS is not yet clear [17]. In the present study, using SOD1^G93A^ mice, we revealed specific and opposite changes in ZnT1 expression at different stages of disease progression. Notably, we observed a significant increase in ZnT1 protein levels early in the disease, without a corresponding increase in ZnT1 mRNA, and lower ZnT1 protein and mRNA levels at later stages compared to wild-type controls. These findings confirm a specific link between the familial SOD1 mutation SOD1^G93A^ and zinc homeostasis alteration [46]. Such evidence is supported by our observations of unaltered ZnT1 protein levels in *in vitro* model of sporadic ALS (L-BMAA neurotoxin). Consistent with this, ZnT3 and ZnT6 were downregulated in the early and late stages of ALS progression in patients with the sporadic form of the disease, whereas no differences were observed in SOD1^G93A^ mice [17]**.** Our results support the concept that familial and sporadic forms of ALS share a common disruption of zinc homeostasis, but the molecular determinants are different. Kim and colleagues [44] report that labile zinc-accumulating cells are undetectable in the spinal cord of SOD1^G93A^ mice up to 14–15 weeks of age and the number of these zinc-accumulating cells increase during disease progression. It is conceivable that zinc efflux mediated by ZnT1 can play a role in those conditions [45]. ZnT1 expression is directly controlled by intracellular zinc concentration [47] and the dual modulation of ZnT1 expression at early and late phases may reflect different transcriptional responses to intracellular zinc levels. The early upregulation of ZnT1 at 10 weeks could represent a homeostatic response to attenuate zinc accumulation, while the reduced expression in the later phase may contribute to motor neuron degeneration due to increased intracellular zinc levels [44]. In line with this, we found that ZnT1 downregulation exacerbates motor neuron damage under neurotoxic conditions. The discrepancy between protein and mRNA levels in the spinal cords of 10-week-old SOD1^G93A^ mice, frequently observed in members of the ZnT family, may reflect differential post-transcriptional regulation of mRNA and protein [48,49]. For the same reasons, the reduction in ZnT1 mRNA and protein levels late in the disease was somewhat unexpected finding and suggested the existence of a post-transcriptional processing that negatively regulates the expression of ZnT1 protein at the mRNA level and blocks its translation. Endogenous miRNAs are potential candidates for this regulation. Through in silico and *in vitro* approaches, we identified and validated miRNA Let-7a as a direct negative regulator of ZnT1. Let-7a was associated with the terminal phase of ALS in our experimental model. Let-7a is one of the oldest and most conserved microRNAs [[50], [51], [52]]. It is widely expressed in the CNS and covers important roles in the physiological and pathological process of the human CNS [53]. In neurological diseases, upregulation of Let-7a has been associated with the induction of inflammatory responses in spinal cord injury (SCI) [54] and Alzheimer's disease [55]. At the same time, its significant downregulation has been found in patients with Parkinson's disease [19] and in sporadic ALS [18]. This negative relationship of the Let-7a/ZnT1 pair appears concordant with our target gene prediction results. Additionally, to strengthen the role of miR let-7a in ALS disease, it has recently been demonstrated that in vivo administration of the antagomir Let-7a promoted nerve regeneration and functional recovery in a rat sciatic nerve transection model [21]. The finding that overexpression of Let-7a in motor neurons triggered cell death confirm that deregulation of such a miRNA could have detrimental effects on cell viability [56]. At the same time, our results extend the role of Let-7a beyond what has been previously described in pathological contests. Importantly, in vivo icv administration of anti-Let-7a early in the symptomatic phase conferred neuroprotection, preserving motor neurons in the lumbar spinal cord and reducing microgliosis and astrogliosis. anti-Let-7a likely counteracted Let-7a upregulation, restoring its physiological levels. This normalization coincided with the restoration of ZnT1 levels both *in vitro* and in vivo and may underline the improved survival and slowed disease progression in SOD1^G93A^ mice.

Although the work focuses on the let-7a/ZnT1 axis, ALS is characterized by complex interactions among numerous genes and signaling pathways [2]. From our results we cannot exclude the possibility that other microRNAs or transporters contribute substantially to zinc deregulation and motor neuron toxicity [57,58]. Additionally, while the intracerebroventricular delivery of anti-let-7a successfully reached the spinal cord neurons in the mouse model, translating this approach to human ALS patients poses significant challenges related to delivery methods, dosing schedules, and long-term safety. Lastly, while the observed improvements in motor function and survival are promising, it remains unclear whether these benefits would remain robust in a larger cohort over extended follow-up periods, and whether they would be as pronounced in ALS models driven by other mutations or sporadic disease mechanisms.

Although we are aware of these limitations, our results support the existence of a novel mechanism involving the ZnT1/Let-7a pair that may be responsible for the downregulation of ZnT1 late in the disease course, which could contribute to increase intracellular zinc levels toward neurotoxic levels.

## Author contributions

S.A. and C.F.,conceived and designed the project in collaboration with G.P., LMT.C. and L.A.; V.V., N.D., N.D., and O.C., performed the experiments; S.A., G.L. and O.C., contributed with animal experiments; S.A., C.F., G.P., LMT.C., and L.A. wrote the manuscript.

## Data and code availability

The data supporting the findings and conclusions of this study are available upon request to the corresponding author, G.P.

## Declaration of competing interest

The authors declare that they have no known competing financial interests or personal relationships that could have appeared to influence the work reported in this paper.
